# Tumor-suppressive function and mechanism of HOXB13 in right-sided colon cancer

**DOI:** 10.1038/s41392-019-0086-1

**Published:** 2019-11-29

**Authors:** Binbin Xie, Bingjun Bai, Yuzi Xu, Yunlong Liu, Yiming Lv, Xing Gao, Fei Wu, Zhipeng Fang, Ying Lou, Hongming Pan, Weidong Han

**Affiliations:** 1 Department of Medical Oncology; Sir Run Run Shaw Hospital; School of Medicine, Zhejiang University, Hangzhou, 310016 PR China; 2 Department of Colorectal Surgery; Sir Run Run Shaw Hospital; School of Medicine, Zhejiang University, Hangzhou, 310016 PR China; 3 Department of Stomatology; Stomatology Hospital; School of Medicine, Zhejiang University, Hangzhou, 310000 PR China; 4 Department of Medical Oncology; The Second Affiliated Hospital of Suzhou University; School of Medicine, Suzhou University, Suzhou, 215000 PR China; 5 School of Medicine, Anhui University of Science and Technology, Huainan, 232001 PR China

**Keywords:** Tumour biomarkers, Tumour biomarkers, Tumour biomarkers, Tumour biomarkers

## Abstract

Right-sided colon cancer (RCC) and left-sided colon cancer (LCC) differ in their clinical and molecular features. An investigation of differentially expressed genes (DEGs) between RCC and LCC could contribute to targeted therapy for colon cancer, especially RCC, which has a poor prognosis. Here, we identified HOXB13, which was significantly less expressed in RCC than in LCC and associated with prognosis in RCC, by using 5 datasets from the Gene Expression Omnibus (GEO). Tissue sample analysis showed that HOXB13 was differentially expressed between normal and only RCC tumor tissues. HOXB13 inhibited colon cancer cell proliferation and induced apoptosis both in vitro and in vivo. Furthermore, we found that HOXB13 might be regulated by DNMT3B and suppress C-myc expression to exert antitumor effects via β-catenin/TCF4 signals in RCC. In conclusion, the current study is the first to demonstrate that HOXB13 has a tumor-suppressive effect in RCC. High expression levels of HOXB13 are associated with prolonged overall survival in patients with RCC. The DNMT3B-HOXB13-C-myc signaling axis might be a molecular target for the treatment of RCC.

## Introduction

The colon is divided into two parts that are derived separately from the embryological midgut and hindgut. One recognized difference between these parts is that the right side of the colon is proximal to the splenic flexure, while the left side of the colon includes the splenic flexure and distal colon.^1,2^ Increasing evidence has demonstrated that right-sided colon cancer (RCC) and left-sided colon cancer (LCC) differ in terms of their clinical, pathological and molecular characteristics.^3–5^ It has been confirmed that RCC is an independent prognostic risk factor.^6,7^ Regarding metastatic colon cancer, different drug sensitivities and different therapeutic efficacies have been observed between RCC and LCC. For example, cetuximab is not recommended in the National Comprehensive Cancer Network (NCCN) guidelines as first-line treatment for metastatic RCC.^8^ The difference in the molecular characteristics of RCC and LCC is thought to explain the poor clinical outcomes of RCC. Previous studies have demonstrated that RCC is more often associated with high microsatellite instability (MSI-H) and KRAS and BRAF mutations than LCC.^9^ Because of the worse prognosis of RCC, there is an urgent need to screen and identify RCC-specific tumor biomarkers for prognosis prediction and to guide the development of potential anticancer therapy.

Here, we used a public genomic database to identify differentially expressed genes (DEGs) according to tumor location and found that HOXB13 is an RCC-specific tumor suppressor gene. HOXB13, a member of the homeobox gene family, is essential for vertebrate embryonic development.^10^ Previous human and animal studies have demonstrated that HOXB13 is often expressed in a tissue-specific and stage-related manner and responsible for body pattern formation.^11,12^ Several studies have shown that HOXB13 plays a crucial role in several types of cancers.^13^ Men with the G84E mutation in HOXB13 have an increased risk of prostate cancer.^14^ Low HOXB13 expression is a poor prognostic factor in patients with gastric cancer.^15^ Compared with its expression in normal colon tissue, HOXB13 expression in colorectal cancer (CRC) is downregulated, a common event in cancer progression.^16,17^ A study based on tumor gene expression data showed that several homeobox genes are related to tumor location.^18^ However, the mechanism of HOXB13, a key gene involved in the localization of colon cancer, and its potential clinical significance remain unclear.

In this study, the prognostic value of HOXB13 and its correlation with the clinical features of RCC and LCC were evaluated based on gene expression profiles in the Gene Expression Omnibus (GEO) database. Then, the results were validated in CRC tumor tissues. Furthermore, cellular and animal experiments were carried out to explore the effect of HOXB13 on tumorigenic processes. Related DEGs and proteins were identified to investigate the mechanism of HOXB13 in RCC. Our results suggest that HOXB13 expression is related to methylation mediated by DNMT3B and has antitumor effects by regulating the expression of C-myc.

## Results

### Analysis of HOXB13 expression in RCC and its prognostic significance based on data from the GEO database

**Fig. 1:**
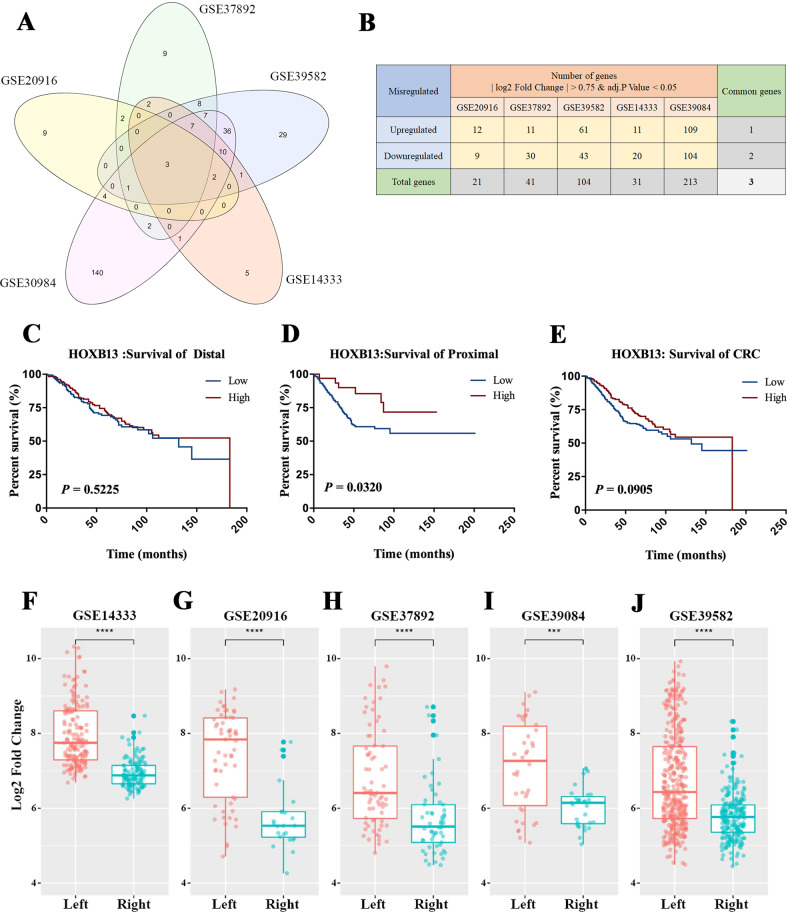
HOXB13 is downregulated and associated with survival in RCC. **a** The intersections of DEGs identified from five datasets (GSE20916, GSE37892, GSE39582, GSE14333 and GSE39084) showed three common differentially expressed genes (DEGs). **b** The numbers of upregulated and downregulated genes among differentially expressed genes in RCC compared to LCC identified from each of the five datasets are shown. Overall survival analyses with data from **c** LCC patients, **d** RCC patients and **e** all CRC patients according to the expression level of HOXB13 were conducted. The expression levels of HOXB13 were compared between left-sided and right-sided CRC patient data in the **f** GSE14333, **g** GSE20916, **h** GSE37892, **i** GSE39084 and **j** GSE39582 datasets. ***P < 0.001, ****P < 0.0001

With a threshold defined by a |log2FC| ≥ 0.75 and an adjusted *P*-value < 0.05, a total of 21, 41, 104, 31, and 213 DEGs according to the location of the CRC (left-sided or right-sided)were identified from the GSE20916, GSE37892, GSE39582, GSE14333 and GSE39084 datasets, respectively. We considered all DEGs identified from each of the five datasets as a collection and identified three DEGs at the intersections of these collections: HOXB13, HOXC6 and PRAC1 (Fig. 1a). Compared with LCC samples, HOXB13 and PRAC1 levels in RCC samples were decreased, while HOXC6 levels in RCC samples were increased (Fig. 1b). The quality of the raw data in each dataset was evaluated by relative logarithmic expression (RLE) and normalized unscaled standard errors (NUSE) analysis (Suppl Fig. 1A–J).

The following clinical data from 566 patients were obtained from the GSE39582 dataset: tumor location, tumor-node-metastasis (TNM) stage and overall survival rate. We performed survival analysis on these samples from different tumor site (right, left or whole colon) based on their high or low expression of these three genes. High HOXB13 expression was associated with better overall survival in only RCC patients (compared with low HOXB13 expression; median survival times were 183 and 132 months, respectively, *P* = 0.032) (Fig. 1c–e). However, high HOXC6 expression was associated with poor overall survival in LCC and all CRC patients but not RCC patients (Suppl Fig. 2A–C). The expression of PRAC1 did not appear to be associated with the survival rates of patients with CRC at any location (Suppl Fig. 2D–F). We then compared the expression levels of HOXB13 in LCC and RCC in each of the 5 datasets. Notably, the expression of HOXB13 in LCC was significantly higher than that in RCC in all five datasets analyzed (Fig. 1f*–*j). Since HOXB13 has prognostic significance in RCC, we speculate that HOXB13 is a key biomarker related to the anatomical site of CRC.

### The relationship between HOXB13 expression and clinicopathologic features

As shown in Table 1, the associations between HOXB13 expression and clinicopathologic features were analyzed in data from 566 patients in the GSE39582 dataset. The expression of HOXB13 was negatively correlated with distant metastasis (*P* = 0.049). In terms of molecular characteristics, patients with low HOXB13 expression were more prone to mismatch repair (MMR) deficiency (*P* = 0.017) and BRAF mutation (*P* = 0.048). Furthermore, high HOXB13 expression was related to a low rate of the positive CpG island methylator phenotype (CIMP). These clinical and molecular features might explain the survival benefit of HOXB13 and signaling pathways involved in this benefit.

**Table1:** Clinicalpathological features according to HOXB13 expression status in 566 CRC patients

	No. patients	HOXB13 Expression		χ^2^	*P* value
		Down-regulation	Up-regulation		
Sex					
Male	310	186	124	1.637	0.201
Female	256	167	89		
Age					
≤65	222	128	94	3.357	0.067
>65	343	224	119		
Location					
Right-sided	224	190	34	**80.74**	**<0.0001**
Left-sided	342	162	180		
Tumor stage					
0 + 1 + 2	301	178	123	2.86	0.091
3 + 4	265	175	90		
T stage					
T0 + Tis + T1 + T2	60	31	29	3.345	0.067
T3 + T4	486	310	176		
Distant Metastasis					
M0	482	293	189	**3.883**	**0.049**
M1	61	45	16		
Lymph node Metastasis					
N0	302	180	122	2.343	0.126
N1 + N2 + N3	244	161	83		
MMR status					
pMMR	444	274	170	**5.67**	**0.017**
dMMR	75	57	18		
CIMP status					
−	405	236	169	**13.72**	**0.0002**
+	91	72	19		
CIN Status					
−	110	74	36	2.234	0.135
+	354	210	144		
TP53 Mutation					
WT	161	97	64	0.061	0.805
M	190	112	78		
Kras Mutation					
WT	328	195	133	2.305	0.129
M	217	143	74		
BRAF Mutation					
WT	461	278	183	**3.992**	**0.048**
M	51	38	13		

Data were analyzed by χ^2^ test. *P* < 0.05 are in bold

### Verification of HOXB13 expression in human tissues

**Fig. 2:**
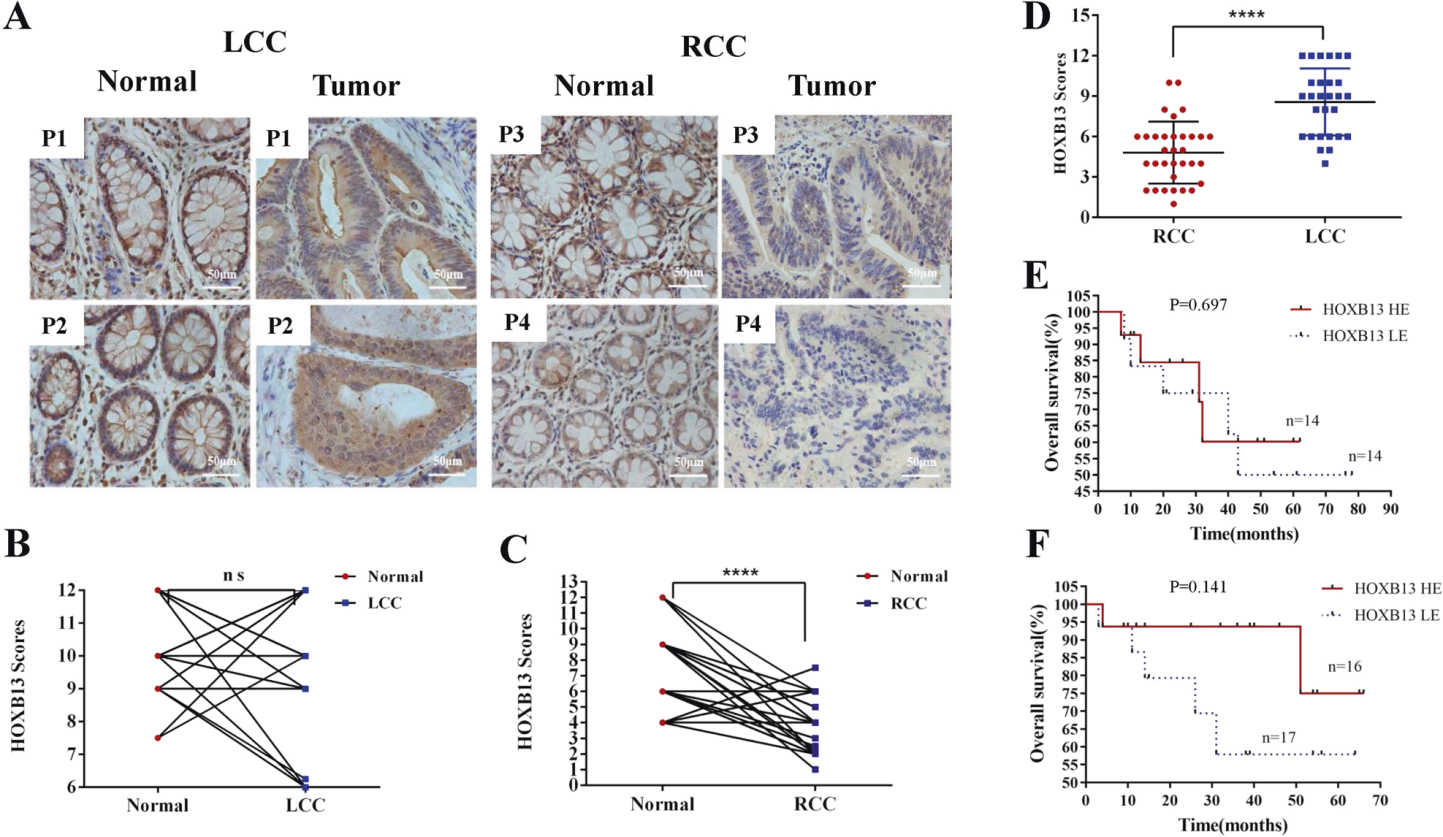
Normal tissues and LCC tissues exhibit higher HOXB13 expression. **a** Representative images showing HOXB13 staining in tumor and normal tissues. Scale bars represent 50 μm. P1-P4, 4 representative patients. **b**, **c** Quantitative analysis of HOXB13 staining scores between LCC and RCC tumor tissues and normal tissues. **d** Quantitative analysis of HOXB13 staining scores in LCC and RCC tissues. Data are presented as the means ± standard deviations (SDs). *P < 0.05, **P < 0.01, ***P < 0.001, ****P < 0.0001. A *t*-test was used for the statistical analysis. Survival analysis of **e** LCC patients (*n* = 28) and **f** RCC patients (*n* = 33) according to HOXB13 staining score. The log-rank (Mantel–Cox) test was used. HOXB13 HE and HOXB13 LE indicate high HOXB13 expression and low HOXB13 expression, respectively

To further verify HOXB13 expression in colon cancer patients and its prognostic significance, we examined the expression of HOXB13 in human tissues from our medical institution. IHC analysis was performed on 61 surgical specimens from patients with sporadic colon cancer to confirm the differential expression of HOXB13 at the protein level. As a control, an antigen depletion study was conducted (Suppl Fig. 3). IHC staining revealed the nuclear localization of the HOXB13 protein in colon samples (Fig. 2a). The expression score of HOXB13 was evaluated by two independent pathologists blinded to the clinical information. In 33 RCC patients, HOXB13 expression in normal samples was higher than that in tumor samples (8.16 ± 0.44 vs 4.82 ± 0.41, respectively, *P* < 0.0001). In LCC patients, there was no difference in HOXB13 expression between tumor and normal tissues (9.05 ± 0.51 vs 10.04 ± 0.28, respectively, *P* = 0.092) (Fig. 2b, c). The expression level of HOXB13 in LCC was significantly higher than that in RCC (9.05 ± 0.51 vs 4.82 ± 0.41, respectively, *P* < 0.0001) (Fig. 2d). To determine the prognostic significance of HOXB13 in patients, patients were divided into a high-level group and a low-level group according to the median IHC scores for HOXB13. The survival rates of RCC and LCC patients were analyzed separately. As shown in Fig. 2e, the survival curves generated from two groups of LCC patients crossed, showing that HOXB13 expression had no effect on prognosis. In RCC (Fig. 2f), patients with high HOXB13 expression achieved a longer overall survival than those with low HOXB13 expression. However, this difference was not statistically significant (*P* = 0.141), which may be because the sample size was small.

In addition to IHC analysis, QPCR was performed. Most of the normal samples presented higher HOXB13 expression than the RCC tumor samples. However, there was no significant difference in HOXB13 expression between LCC tumor samples and normal samples (Suppl Fig. 4A, B). Compared with those in LCC, HOXB13 mRNA levels in RCC were significantly lower (505.1 ± 140.2 vs 220 ± 80.4, respectively, *P* = 0.009). Western blotting also showed the same results at the protein level, indicating that HOXB13 expression was upregulated in LCC compared with RCC (Suppl Fig. 4C, D).

### Antitumor effect of HOXB13 in vitro

**Fig. 3:**
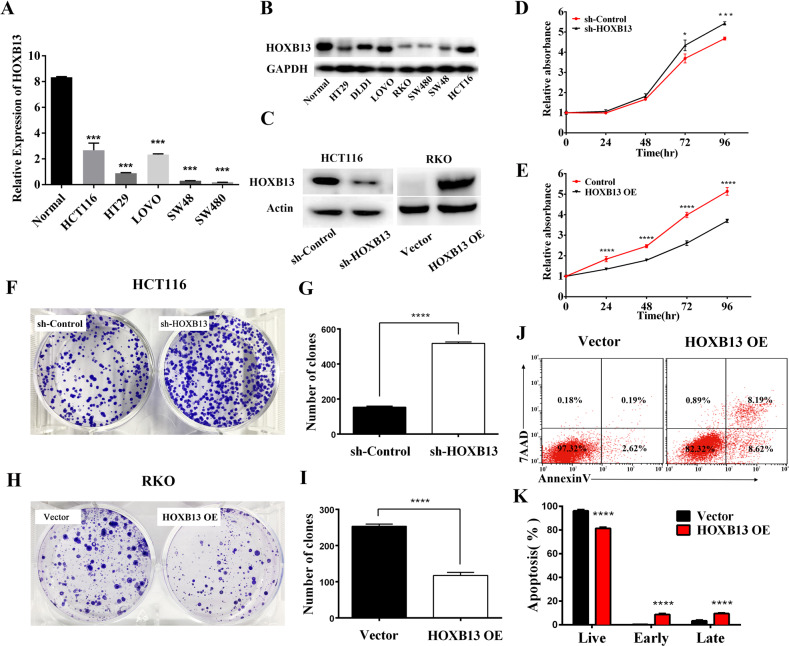
HOXB13 exhibits antitumorigenic properties in colon cancer cells. **a** The mRNA levels of HOXB13 in human colon cancer cell lines and a normal human colon mucosal epithelial cell line were compared. **b** Protein levels of HOXB13 were examined by western blotting. GAPDH was used as a loading control. **c** HOXB13 knockdown and overexpression were validated by western blot analysis. **d**, **e** A CCK-8 assay was performed to assess the proliferation of HCT116/sh-HOXB13 and RKO/HOXB13 OE cells. **f**–**i** A cell clonogenic assay was used to measure the capacity of HCT116/sh-HOXB13 and RKO/HOXB13 OE cells to form colonies. Data are presented as the mean ± SD. ****P < 0.001. A t-test was used for the statistical analysis. **j**, **k** Flow cytometry and data analysis were performed to examine apoptosis in RKO/HOXB13 OE cells

To address the biological function of HOXB13, we used lentiviral infection to knock down or overexpress HOXB13 in CRC cell lines. First, the expression of HOXB13 was detected in human colon cancer cell lines (DLD1, RKO, HCT116, HT29, SW48, SW480, and LOVO cells) and a normal human colon mucosal epithelial cell line. As shown in Fig. 3a, b, HOXB13 mRNA and protein expression was significantly decreased in colon cancer cell lines compared to normal control cells (*P* < 0.001). Among the seven cell lines tested, RKO cells exhibited the lowest expression of HOXB13, while HCT116 cells exhibited the highest expression of HOXB13. Therefore, HCT116 cells were chosen to construct a knockdown cell line (HCT116/sh-HOXB13), with control shRNA-transfected cells (HCT116/sh-Control) used as a control. In addition, RKO cells were chosen to construct an HOXB13 overexpression cell line (RKO/HOXB13 OE), with empty vector-transfected cells (RKO/Vector) as a control (Fig. 3c). As shown in Fig. 3d, e, cell proliferation was promoted by the downregulation of HOXB13 and inhibited by the overexpression of HOXB13. In addition, the knockdown of HOXB13 enhanced colony formation, while the overexpression of HOXB13 significantly reduced colony formation (Fig. 3f–i). Flow cytometry analysis showed that HOXB13 overexpression increased both early and late-stage apoptosis (Fig. 3j, k). Moreover, the wound-healing assay indicated that the downregulation of HOXB13 promoted cell migration, while the upregulation of HOXB13 had the opposite effect, suggesting that HOXB13 attenuates cell migration in colon cancer cells (Suppl Fig. 5A–D). The results of Transwell chamber assays were also consistent with those of the wound-healing assay, indicating the inhibitory effect of HOXB13 on cell migration and invasion (Suppl Fig. 5E–L).

### Antitumor effect of HOXB13 in vivo

**Fig. 4:**
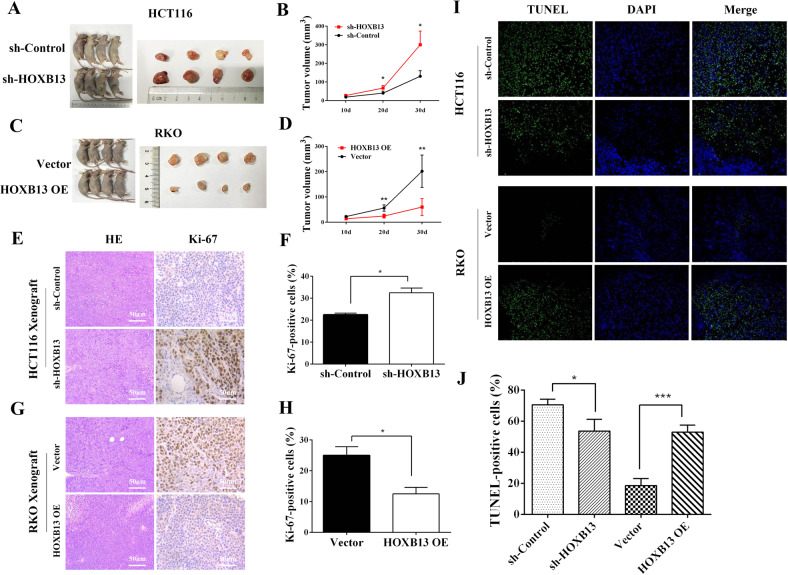
HOXB13 exhibits antitumorigenic properties in colon cancer cells in vivo. **a**–**d** Representative images of euthanized mice and xenograft tumors at day 30 after subcutaneous injection (*n* = 6). Tumor sizes are depicted as the tumor volume. **e**, **g** Representative images showing HE and Ki-67 staining of xenograft tumor tissues. Scale bars represent 50 μm. **f**, **h** Comparison of the proportion of Ki-67-positive HCT116/sh-HOXB13 (or RKO/HOXB13 OE) cells and the corresponding controls. **i**, **j** Apoptotic cells in xenograft tumors were detected with TUNEL assays. Data are presented as the means ± SDs. *P < 0.05, **P < 0.01, ***P < 0.001

To verify the suppressive effect of HOXB13 on the progression of colon cancer in vivo, we constructed a xenograft tumor model using HOXB13 knockdown and HOXB13-overexpressing CRC cells. Nude mice inoculated with HCT116/sh-HOXB13 cells had an increased tumor load compared with control shRNA-transfected xenograft tumors at 20 days (67.5 ± 6.6 vs 40 ± 5.4, respectively, *P* < 0.05) and 30 days (300 ± 36.74 vs 130.8 ± 15.28, respectively, *P* < 0.05) after inoculation (Fig. 4a, b). Conversely, overexpression of HOXB13 significantly inhibited CRC growth (Fig. 4c, d). These data indicate that HOXB13 acts as a tumor suppressor and negatively regulates tumor growth. Tumor tissues from nude mice were then used for hematoxylin-eosin (HE) staining and IHC analysis. Compared with control tumors, HCT116/sh-HOXB13 tumors were more aggressive with the following characteristics: decreased differentiation and increased atypia and mitosis counts. More intense Ki-67 staining was observed in tumor cells with low HOXB13 expression compared to control cells (32.5 ± 1.5 vs 22.5 ± 0.5%, respectively, *P* = 0.024) (Fig. 4e–h). The opposite result was observed when RKO/HOXB13 OE cells were compared to their parental control cells. TUNEL assays indicated that the overexpression of HOXB13 promoted apoptosis in xenograft tumors, while the knockdown of HOXB13 prevented cell apoptosis (Fig. 4i, j). Western blotting of xenograft tumors showed that HOXB13 overexpression upregulated cleaved-PARP, P53, and BAX and downregulated BCL-2 and C-myc (Suppl Fig. 6A, B). Collectively, the results of the animal study further confirmed the antitumor properties of HOXB13 in colon cancer.

### Identification of apoptosis-related proteins

**Fig. 5:**
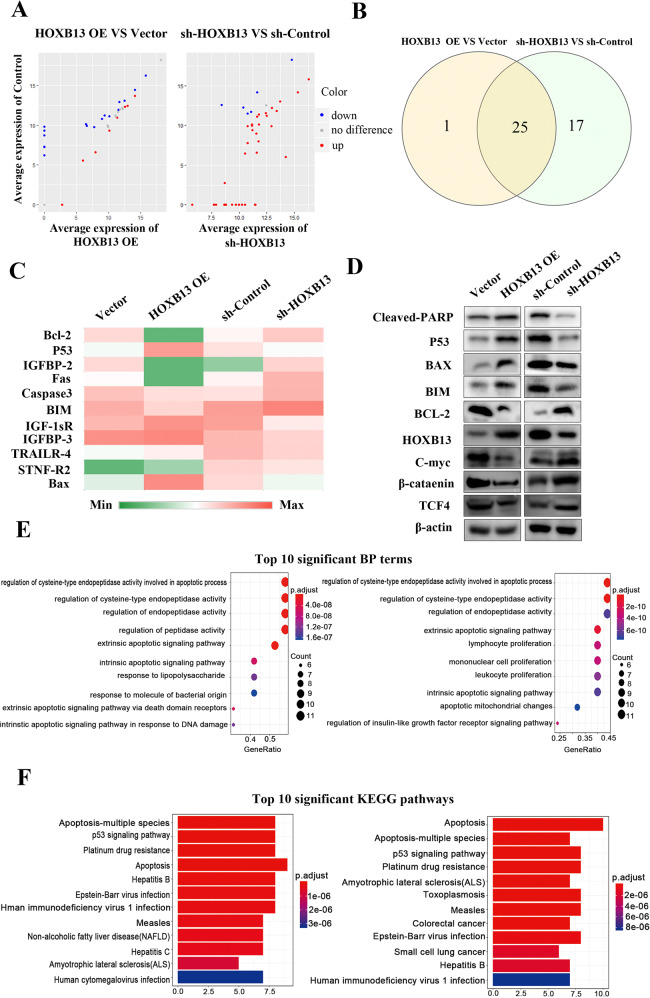
HOXB13 regulates apoptosis-related proteins in colon cancer cells. **a** A human apoptosis antibody array was used to detect the expression of apoptosis-related proteins in two pairs of cells (HCT116/sh-HOXB13 and RKO/HOXB13 OE cells and the corresponding controls). A scatter diagram was drawn to show the average expression of proteins in HCT116/sh-HOXB13 and RKO/HOXB13 OE cells compared to the corresponding controls. **b** Intersection of differentially expressed proteins between the two pairs of cells. **c** Heat map showing the expression levels of representative differentially expressed proteins from the array data. The color indicates the level of protein expression (low expression (green) and high expression (red)). **d** Western blotting was performed to detect differentially expressed proteins between the two pairs of cells. **e**, **f** GO functional and KEGG analyses were performed to investigate the biological processes (BP) and pathways enriched in HOXB13-related DEGs. The top ten most significantly enriched BP terms and KEGG pathways are shown

Our in vitro results showed that HOXB13 promotes cell apoptosis. To investigate apoptosis-related proteins, an analysis was performed with Human Apoptosis Proteome Profiler Array. HCT116/sh-HOXB13 versus sh-Control and RKO/HOXB13 OE cells versus vector-transfected cells were divided into two groups, and 15 differentially expressed proteins between the groups were identified (Fig. 5a, b). As shown in Fig. 5c, IGF-1sR and insulin-like growth factor-binding protein (IGFBP), representative apoptotic markers and members of the insulin like growth factor (IGF) family, were differentially expressed. Moreover, regulation of the Bcl-2 family members Bax, Bcl-2, and Bim indicated the involvement of the mitochondrial pathway in apoptosis. Changes in the expression of these apoptosis-related proteins were validated by western blot analysis. As shown in Fig. 5d, the expression of the proapoptotic protein cleaved-PARP and the tumor suppressor P53 decreased, while expression of the oncogenic protein C-myc increased in HOXB13 knockdown HCT116 cells. In contrast, overexpression of HOXB13 in RKO cells increase the expression of cleaved-PARP and P53 and decreased the expression of C-myc. Functional analysis revealed that most of the DEGs were enriched in the “regulation of peptidase activity”, “apoptotic signaling pathway” and “immune cell proliferation” GO terms (Fig. 5e). Furthermore, most of the DEGs were enriched in the “apoptosis”, the “P53 signaling pathway” and “platinum drug resistance” KEGG pathways (Fig. 5f).

### DNMT3B-HOXB13-C-myc signaling axis in CRC

**Fig. 6:**
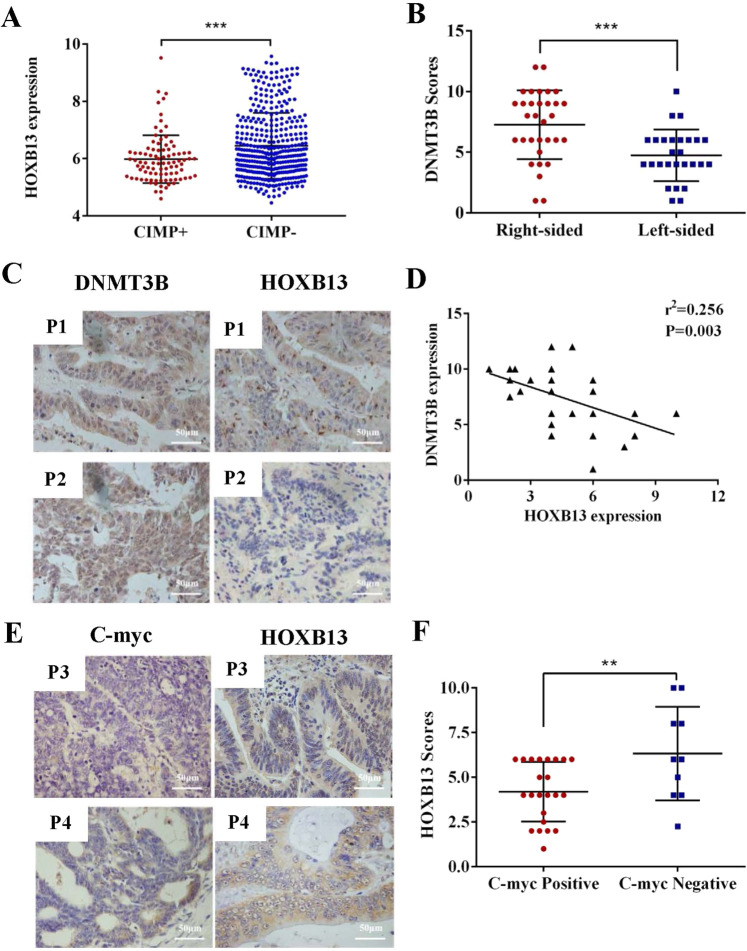
HOXB13 expression is downregulated by DNMT3B, which inhibits C-myc expression in RCC. **a** Analysis of the relationship between HOXB13 and CIMP status based on the GSE39582 dataset. **b** Comparison of DNMT3B IHC scores in RCC (*n* = 33) and LCC (*n* = 28). **c** Representative images showing HOXB13 and DNMT3B staining in RCC tissues. Scale bars represent 50 μm. **d** Quantitative analysis of the correlation between HOXB13 and DNMT3B expression in RCC (*n* = 33) according to IHC staining score. **e** Representative images showing HOXB13 and C-myc staining in RCC tissues. **f** Comparison of HOXB13 and C-myc expression in RCC according to IHC scores (*n* = 33). P1–P4, 4 representative patients. Data are presented as the means ± SDs. **P < 0.01, ***P < 0.001

As shown in Table 1 and Fig. 6a, clinical information from the databases revealed that CIMP was associated with HOXB13 expression (*P* = 0.0002). The DNMT3B gene, which encodes a major type of DNA methyltransferase, functions in de novo methylation.^19^ We used human tissues to explore the correlation between HOXB13 and DNMT3B expression in RCC. The IHC results showed that the expression of DNMT3B in RCC was higher than that in LCC (7.27 ± 0.50 vs 4.74 ± 0.41, respectively, *P* = 0.0003) (Fig. 6b). Linear regression analysis of the IHC scores for HOXB13 and DNMT3B showed that HOXB13 was negatively correlated with DNMT3B in RCC (*r*^2^ = 0.256, *P* = 0.003) (Fig. 6c, d). Linear regression analysis also revealed a negative correlation between HOXB13 and DNMT3B mRNA in RCC (*r*^2^ = 0.537, *P* = 0.0002) (Suppl Fig. 7A). However, according to their IHC scores, no such correction was found in LCC (*r*^2^ = 0.021, *P* = 0.474) (Suppl Fig. 7B).

To elaborate on the relationship between DNMT3B and HOXB13, we used lentiviral infection to knock down or overexpress DNMT3B in the HCT116 and RKO cell lines, and the efficiency of DNMT3B knockdown and overexpression was assessed by western blotting. As shown in Supplementary Fig. 8A, B, the knockdown of DNMT3B upregulated HOXB13 expression in the two cell lines, while HOXB13 was downregulated in the DNMT3B overexpression cell lines, which was consistent with the aforementioned IHC results. Then, HCT116 and RKO cells were treated with the DNA methyltransferase inhibitor decitabine to inhibit methylation. As expected, the expression of DNMT3B decreased, while that of HOXB13 increased in a time-dependent manner (Suppl Fig. 8C). To further explore the regulatory effect of DNMT3B on HOXB13, we performed methylated DNA immunoprecipitation analysis (MedIP) and measured enrichment in the MedIP fraction by PCR. Supplementary Fig. 8D shows that methylation of the HOXB13 promoter was decreased after the downregulation of DNMT3B but increased when DNMT3B was upregulated. These results revealed that DNMT3B regulates HOXB13 expression levels through promoting the DNA methylation of the CpG island of HOXB13.

We further examined the biological function of DNMT3B in colon cancer. DNMT3B knockdown (RKO/sh-DNMT3B) and overexpression (HCT116/DNMT3B OE) cell lines were used for the in vitro experiment. As shown in Supplementary Fig. 9A, B, cell proliferation and colony formation were dramatically enhanced in HCT116/DNMT3B OE cells but impaired in RKO/sh-DNMT3B cells. Next, the impact of DNMT3B knockdown or overexpression on cell migration was assessed by an independent assay based on coated Transwell chambers. We found that DNMT3B overexpression significantly increased cell migration in HCT116 cells, while RKO/sh-DNMT3B cells exhibited inhibited cell migration (Suppl Fig. 9C). These findings established that DNMT3B promotes the proliferation and migration of colon cancer cells.

Our in vitro experiment showed that the upregulation of HOXB13 was accompanied by the downregulation of C-myc. To further validate this finding in clinical samples, RCC tissues were stained for HOXB13 and C-myc. As shown in Fig. 6e, patients with high HOXB13 expression had low C-myc expression, although C-myc is known to be expressed at high levels in tumor tissues. IHC scoring showed that the expression of HOXB13 in patients negative for c-myc expression was higher than that in patients that positively expressed c-myc (*P* < 0.01) (Fig. 6f). In addition, QPCR showed that HOXB13 was negatively correlated with C-myc at the mRNA level (*r*^2^ = 0.321, *P* = 0.007) (Suppl Fig. 7C). However, no significant correlation was found between the IHC scores for HOXB13 and C-myc in the LCC samples (Suppl Fig. 7D). Consistently, the mRNA levels of HOXB13, DNMT3B and C-myc were not significantly correlated in LCC (data not shown).

### Expression patterns of DNMT3B, HOXB13, and C-myc in CRC

**Fig. 7:**
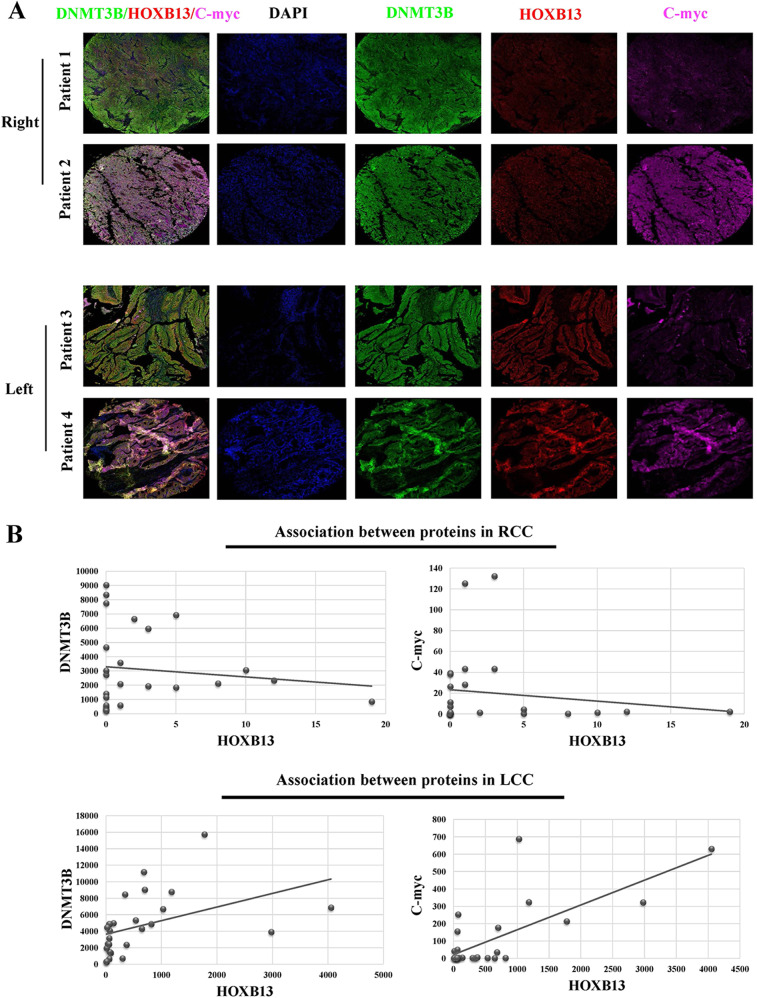
HOXB13 expression is negatively correlated with DNMT3B and C-myc expression in RCC. **a** Representative fluorescence images showing DNMT3B, HOXB13 and C-myc staining in CRC tissues; each tumor slide was stained for DNMT3B, HOXB13 and C-myc, which are indicated by the green, red and pink fluorescence channels, respectively. DAPI was used to visualize nuclei. **b** Quantification of DNMT3B +, HOXB13 + and C-myc + cells in CRC samples. Linear regression was performed to analyze correlations between the levels of different proteins in RCC and LCC

To verify the expression patterns of DNMT3B, HOXB13, and C-myc, multiplex immunohistochemistry (mIHC) was performed with tissue microarrays (TMAs) containing 29 RCC and 27 LCC samples. Each slide was simultaneously stained for DNMT3B, HOXB13 and C-myc. Four representative images, two from RCC patients and two from LCC patients, are shown in Fig. 7a. The three proteins were expressed in the nuclei of tumor cells. High levels of DNMT3B and C-myc expression and low levels of HOXB13 expression were detected in RCC patients. However, this correlation was not detected in LCC patients. Statistical analysis showed that the average number of HOXB13-positive cells in the LCC samples was significantly higher than that in the RCC samples (594 vs 2 cells per mm^2^, respectively). Linear regression analysis revealed that the number of HOXB13-positive cells was negatively correlated with the number of DNMT3B- and C-myc-positive cells in RCC (Fig. 7b). However, this negative correlation between HOXB13 and DNMT3B or C-myc was not established in LCC.

## Discussion

One of the major differences between RCC and LCC is their difference in gene expression.^20^ Key genes affect clinical characteristics, treatment strategies and prognosis.^21^ Because of the poor prognosis of RCC, the need to find causal molecular events in RCC to help develop precise treatment is urgent. In the present study, gene expression information from 5 GEO datasets and clinical tumor tissues showed that HOXB13 is differentially expressed in RCC and LCC and has prognostic significance in only RCC. We identified HOXB13 as a tumor suppressor gene in RCC and suggest the DNMT3B-HOXB13-C-myc signaling axis as a potential target pathway for the treatment of this highly malignant disease.

Previous studies have investigated DEGs between RCC and LCC using gene chips and patient samples or gene expression information from databases.^22–24^ In the present study, DEGs at the intersection of 5 datasets from the GEO were identified, and HOXB13, which has a unique effect on other genes, was the focus of this study because of its impact on overall survival in RCC. Furthermore, analysis of HOXB13 expression at the mRNA and protein levels in human tissue samples verified its differential expression between RCC and LCC, as shown by data from the database. Additionally, HOXB13 was revealed to be downregulated in only RCC tumor tissue compared to normal tissues. The use of multiple datasets and human tissues to identify key genes between RCC and LCC produced more comprehensive and reliable results. In summary, cellular and animal experiments have shown that HOXB13 may be an important tumor suppressor gene and that low HOXB13 expression plays a critical role in the pathogenesis and progression of RCC. study result went one step further than those of Tatangelo^25^ and Ghoshal.^26^ Tatangelo confirmed the aberrant expression of HOXB13 in RCC and LCC without investigating the biological function of this difference in expression. Our results were in contrast to Tatangelo’s finding that HOXB13 is downregulated in normal tissue compared to tumor tissue, which might be explained by differences in the study populations and specimens. In addition, Ghoshal revealed the tumor-suppressive effect of HOXB13 but did not determine the effect of HOXB13 on the anatomical location of colorectal cancer.

Although HOXB13 has been associated with CRC, our results showed that patients with high levels of HOXB13 expression had a better prognosis than those with low levels of HOXB13, but this difference was found in only RCC. HOXB13 was not found to have prognostic value in patients with both types of CRC or LCC. Previous studies have identified some genes that exhibit a tumor location-dependent correlation with prognosis. For example, NADPH oxidase 4 (NOX4) was found to be a predictive factor for relapse in stage II LCC, whereas integrin alpha 3 beta 1 (ITGA3) plays a role in predicting relapse in stage II RCC.^27^ Gene subtypes might also be associated with drug sensitivity, depending on the location of the tumor. The latest NCCN clinical practice guidelines for colon cancer state that panitumumab or cetuximab should be used as a first-line treatment for only KRAS wild-type LCC among metastatic CRCs.^8,28^ According to the results of the present study, the biological function and prognostic value of HOXB13 might apply to only RCC. Clinical information from the database showed that HOXB13 expression is related to MMR status and BRAF mutation. These factors are molecular characteristics that affect overall survival. However, it is difficult to explain why the role of HOXB13 in predicting prognosis is location-dependent.

The methylation statuses of RCC and LCC differ considerable. The CIMP is characterized by poor prognosis and associated with BRAF mutation, high MSI, and a right-sided tumor location.^29–31^ In this study, analysis of clinical information from the GEO database showed that more RCC than LCC patients had a positive CIMP status and that CIMP status was related to the downregulation of HOXB13. Currently, hypermethylation is thought to be one of the most common mechanisms by which tumor suppressor genes are downregulated in cancer cells.^32,33^ In different types of cancers (renal cell carcinoma, prostate cancer and malignant melanoma), HOXB13 is methylated at an upstream CpG island.^34–36^ DNMT3B, a DNA methyltransferase, is associated with the CIMP and may play a causal role in tumorigenesis.^37^ In the current study, DNMT3B was used as a biomarker to measure the methylation status of CRC tissue. Hypermethylation was shown to cause the downregulation of HOXB13 and worsen clinical outcome, consistent with the conclusions of Ghoshal in CRC.^26^ Our results further suggest that the HOXB13 methylation is regulated by DNMT3B in only RCC.

To determine the molecular events of HOXB13 in tumorigenesis, C-myc was detected in human tissue. The myc family consists of proto-oncogenes encode transcription factors that play important roles in regulating cell growth, apoptosis and differentiation.^38,39^ C-myc is a target gene of the Wnt signaling pathway and activated by the β-catenin/TCF4 complex.^40^ C Jung et al.^16^ reported that HOXB13 downregulates TCF4 and C-myc to inhibit β-catenin/TCF-mediated signaling and subsequent tumor growth, which is consistent with our conclusions. In the present study, the expression of C-myc in RCC was negatively correlated with the mRNA and protein levels of HOXB13. According to in vitro western blotting and in vivo experiments, the expression of β-catenin and TCF4 was increased in HOXB13 knockdown cells compared to control cells (Fig. 5d and Suppl Fig. 6A). The results above indicated that the DNMT3B-induced downregulation of HOXB13 mediates methylation and regulates C-myc expression via β-catenin/TCF4 signaling, enhancing tumor progression and resulting in a poor prognosis. The DNMT3B-HOXB13-C-myc pathway was shown to play an essential role in RCC. Although the direct or indirect regulatory relationship between these proteins has not been elucidated by more precise experiments, our findings suggest a potential novel therapy for RCC. The DNMT inhibitor SGI-1027, the safety of which was validated in a rat hepatoma (H4IIE) cell line, decreased methylation by inhibiting the activity of DNMT3B.^41^ This DNMT inhibitor might upregulate HOXB13 expression by decreasing methylation in cancer cells. C-myc inhibitors have been widely used in vivo and in vitro to induce cycle arrest and apoptosis in cancer cells and enhance chemosensitivity.^42,43^ Further studies of these inhibitors as antitumor agents in colon cancer, especially in RCC, might be valuable.

**Fig. 8:**
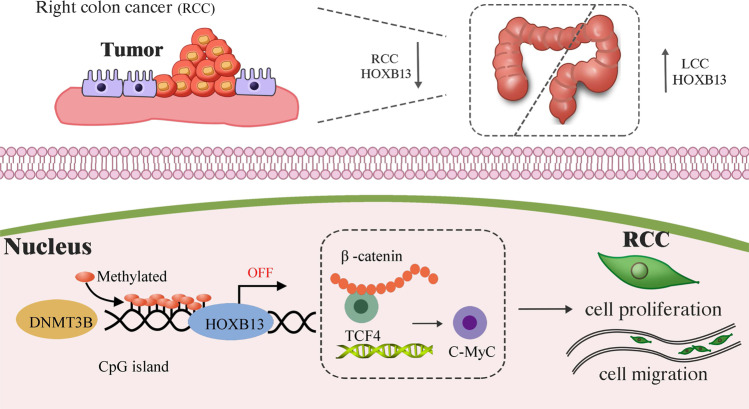
A model showing the role of HOXB13 in RCC. HOXB13 is downregulated in RCC tumor tissue compared to normal tissue and LCC tumor tissue. HOXB13 downregulation in RCC is caused by the DNMT3B-induced methylation of an upstream CpG island, which increases C-myc expression by activating the β-catenin/TCF4 complex, thus enhancing tumor cell proliferation and migration

In conclusion, the present study confirms that HOXB13 can inhibit RCC by regulating multiple pathways related to apoptosis, proliferation and migration. High expression levels of HOXB13 are predictive of a good prognosis in RCC, which can be explained by the regulation of DNMT3B-mediated methylation by HOXB13, and the subsequent regulation of C-myc expression through β-catenin/TCF4 signaling (Fig. 8). Our results highlight the role of tumor localization in the prognostic value of genes. The DNMT3B-HOXB13-C-myc axis is a potential signaling pathway for molecule-targeted therapy in RCC. However, tumor location is difficult to investigate in cellular and animal models. Further studies in animal models based on specific tumor locations can provide more reliable evidence to support the role of HOXB13 in RCC.

## Materials and methods

### Microarray data and survival analysis

The GEO (http://www.ncbi.nlm.nih.gov/geo) is a freely available, public repository of microarray and next-generation sequencing data. Five gene expression profiles (GSE20916, GSE37892, GSE39582, GSE14333 and GSE39084) were obtained from the GEO database. These datasets containing expression data from tumor samples with the corresponding tumor location were based on the GPL570 platform and collected by Affymetrix Human Genome U133 Plus 2.0 arrays. A total of 1092 tumor samples were contained in these five profiles. After their download, we analyzed the data with the R (edgeR) package (http://bioconductor.org/packages/release/bioc/html/edgeR.html), which was used to obtain DEGs according to the conditions of a log2 fold change (log2FC) ≥ 0.75 and an adjusted *P*-value < 0.05. These DEGs were evaluated by Student’s *t*-test. The Kaplan–Meier (KM) method was performed for survival analysis. A log-rank test was used to compare the survival distributions, and differences with *P* < 0.05 were considered significant. All analyses were performed on the R 3.5.0 framework. Survival curves were plotted in GraphPad Prism (version 7, GraphPad, Inc.).

### Collection of fresh tissue samples

This study was approved by the Sir Run Run Shaw Hospital and Zhejiang University Ethics Committee. Fresh tumor tissues and adjacent normal tissues (5.0 cm beyond the tumor tissues) were obtained directly from the operation specimens of 61 consecutive patients who underwent surgical resection for primary sporadic CRC at the Department of Colorectal Surgery, Sir Run Run Shaw Hospital, Hangzhou, Zhejiang, China, between January 2014 and January 2015. Written informed consent for tissue collection was obtained from all patients prior to their surgical procedure. The adjacent normal tissues were collected from more than 5 cm away from the cancerous tissue.

### Quantitative real-time PCR (QPCR)

Fresh tissue samples (100 μg) were homogenized using a grinder with liquid nitrogen, followed by lysis with TRIzol reagent (Invitrogen Beijing Office, Beijing, China). RNA was quantified on a NanoDrop 2000c spectrophotometer (Thermo Fisher Scientific, Inc., Waltham, MA, USA). cDNA was synthesized using an RNeasy Mini Kit (TakaRa, Shimogyo-ku, Kyoto, Japan). QPCR analysis was performed with SYBR Green Master Mix (TaKaRa) on an ABI 7500 sequence detector under the following conditions: 95 °C for 5 min; 45 cycles of 95 °C for 5 s and 60 °C for 30 s; one cycle of 95 °C for 5 s, 60 °C for 1 min and 95 °C for 15 s; and finally 50 °C for 30 s. Sequences of the primers used are listed in Supplementary Table 1. β-Actin was used as an internal control gene. Relative expression was analyzed using the 2−ΔΔCq method. Data were normalized and are expressed as the fold change relative to the control sample.

### Western blot analysis

Whole-cell lysates were prepared using RIPA buffer [10 mM Tris–HCl (pH 7.4), 150 mM NaCl, 1% NP-40, 1 mM EDTA, and 0.1% SDS], separated by SDS-PAGE, and transferred onto polyvinylidene difluoride (PVDF) membranes (Millipore, Belfor, MA, USA). The membranes were then incubated overnight at 4 °C with antibodies against HOXB13 (Sangon, Shanghai, China; cat. no. D162419, 1:1000 dilution), DNMT3B (HuaAn Biotechnology Company, ET1605-9, 1:1000 dilution) and GAPDH (Cell Signaling Technology, Inc., Danvers, MA, USA; cat. no. 5174, 1:1000 dilution). After washing in Tris-buffered saline containing Tween 20 (TBST), the membranes were incubated for 2 h in horseradish peroxidase (HRP)-conjugated secondary antibodies (Sangon, Shanghai, China; cat. no. D110058-0100, 1:5000 dilution) at room temperature. Excess secondary antibodies (Sangon, Shanghai, China; cat. no. D110058-0100, 1:1,0000 dilution) were and rinsed from the membranes by washing with TBST. Signals were visualized with an enhanced chemiluminescence kit (Biological Industries, Kibbutz Beit Haemek, Israel).

### Immunohistochemistry (IHC)

Tissue samples were collected from CRC tumor surgical specimens. A standard IHC protocol was employed with all formalin-fixed, paraffin-embedded samples to evaluate the expression of HOXB13 in CRC. Tissue sections were cut into 4-μm-thick slices from paraffin blocks, deparaffinized, dehydrated, and subjected to antigen retrieval. The slices were incubated with a primary antibody against HOXB13 (dilution 1:300, cat. no. ab28575, Abcam, Cambridge, UK) for 1 h in a humidified chamber. Then, a PV‐9000 two‐step immunohistochemical staining kit (Zhongshan Jinqiao Biotechnology Co., Ltd., Beijing, China) was used, and 3,3′‐diaminobenzidine tetrahydrochloride (DAB) staining and hematoxylin counterstaining were carried out according to the manufacturer’s protocol.

### Cells culture and treatment with decitabine

Human colon cancer cell lines (DLD1, RKO, HCT116, HT29, SW48, SW480, and LOVO cells) and a normal human colon mucosal epithelial cell line were purchased from the Institute of Cell Research in Shanghai, China. Cells were maintained in RPMI-1640 medium supplemented with 10% fetal bovine serum (FBS) and cultured in a humidified atmosphere with 5% CO_2_. All cultures were given fresh medium every 3–4 days. RKO and HCT116 cells were treated with 2.5 μM decitabine (Selleckchem, Houston, TX, USA) for 24–72 h.

### Cell transfection and stable colony selection

To inhibit HOXB13 expression in HCT116 cells, the following three short hairpin RNA (shRNA) sequences targeting HOXB13 were designed (5′-3′): CCGGCGCCAGATTACCATCTGGTTTCTCGAGAAACCAGATGGTAATCTGGCGTTTTTG, CCGGGTTTGCCTTCTATCCGGGATACTCGAGTATCCCGGATAGAAGGCAAACTTTTTG, and CCGGCCCGTGCCTTATGGTTACTTTCTCGAGAAAGTAACCATAAGGCACGGGTTTTTG. The first sequence was cloned into the lentiviral vector GV248, which was then transfected into HCT116 cells to construct the HOXB13 knockdown cell line (HCT116/sh-HOXB13). HOXB13 cDNA was cloned into the lentiviral expression vector GV492 (GeneChem, Shanghai, China) and then transfected into RKO cells to construct the HOXB13 overexpression cell line (RKO/HOXB13 OE). Transfection was performed using Lipofectamine 2000 (Invitrogen, USA) following the manufacturer’s guidelines. HOXB13 knockdown and overexpression were validated by western blot analysis. The same method was used to construct DNMT3B knockdown and overexpression colon cancer cell lines. The following shRNA sequence targeting DNMT3B was designed: (5′-3′): CCGGGCCTCAAGACAAATTGCTATACTCGAGTATAGCAATTTGTCTTGAGGCTTTTT.

### Cell proliferation

Cell proliferation was measured using Cell Counting Kit-8 (CCK-8; Dojindo, Tokyo, Japan) according to the manufacturer’s instructions. Transfected cells and the corresponding controls were seeded into 96-well plates and cultured for 24, 48, 72, and 96 h. The optical density (OD) was measured at 450 nm using a microplate reader (Thermo LabSystems, Helsinki, Finland). All experiments were performed in triplicate.

### Cell clonogenic assay

In total, 2000 cells were cultured in 6-cm dishes in normal culture medium and incubated in an incubator containing 5% CO_2_ at 37 °C for 14 days. Individual colonies (>50 cells per colony) were fixed and stained with a solution containing 0.25% crystal violet stain. The colonies were photographed and counted. Each experiment was performed in triplicate and repeated twice.

### Cell migration analysis

Cells were cultured in 6-cm dishes in RPMI-1640 medium supplemented with 10% FBS overnight. The cells were then scratched with a pipette tip (0 h) and cultured in complete RPMI-1640 medium. Images were taken at 0 h and 24 h after wounding. The migration rate was calculated by measuring the change in wound width.

### Transwell assay

A 24-well Transwell culture chamber with 8 μm PET membranes (Millipore, Bedford, MA, USA) was used to assess cell migration. Cells were trypsinized at room temperature and later centrifuged at 800 rpm for 5 min. After being resuspended in serum-free RPMI-1640 medium, 100 µl of cell suspension (2–5 × 10^4^) was seeded into each upper invasion chamber, and 500 µl of medium containing 10% FBS was added to the lower chamber. Cells were maintained for 12–24 h at 37 °C in a 5% CO_2_ incubator. Afterwards, cells in the upper chamber were scraped away, and migrated cells on the lower surface were fixed with ethanol and stained with 0.05% crystal violet. Images of ten random high-power fields were captured with a digital camera, and the number of migrated cells was determined. Migrated cells in five random visual fields were counted under an inverted microscope (×200).

### Flow cytometry analysis

To confirm the effect of HOXB13 on apoptosis, an Annexin FITC/7-AAD Apoptosis Detection Kit was used. Briefly, cells (1 × 10^5^ cells/well) were harvested, washed twice with phosphate-buffered saline (PBS), and then resuspended in 500 μl of 1 × binding buffer provided in the kit. The cells were incubated in the dark at room temperature with 5 µl of an Annexin V solution and 5 µl of a 7AAD solution for 5 min. Apoptosis levels were analyzed using flow cytometry (BD, USA), and the data were analyzed with FlowJo 10.0.5 (Tree Star, Inc., Ashland, OR, USA) software.

### Xenograft tumor model

The animal experiments were approved by the Sir Run Run Shaw Hospital Institutional Animal Care and Use Committee and conducted according to the Guide for the Care and Use of Laboratory Animals of Zhejiang University. Nude mice were purchased from the Institute of Animal Research in Shanghai, China. All mice were housed, handled, and euthanized in accordance with federal and institutional guidelines under supervision of the Ethics Committee of the Sir Run Run Shaw Hospital, Zhejiang University. Four-week-old nude female mice were randomly divided into 4 groups (sh-HOXB13, sh-Control, HOXB13 overexpression (OE) and vector, *n* = 6 per group) and injected subcutaneously with 2 × 10^6^ cells (100 μl) into the right axilla. The length (L) and width (W) of the xenograft tumors were measured every 10 days, with the W smaller than the L. The xenograft volume (V) was calculated as V = (L × W^2^)/2. After 30 days, the tumors were collected and photographed.

### TdT-mediated dUTP nick-end labeling (TUNEL) assay

Tumor sections were obtained from nude mice, deparaffinized, hydrated, and incubated with Proteinase K for 15 min. After being washed in PBS 4 times, the sections were analyzed with a TUNEL In Situ Cell Death Detection Kit according to the manufacturer’s instructions (Sigma-Aldrich). Then, the sections were counterstained with DAB (Dako). The total number of TUNEL-positive cells in 10 nonoverlapping random fields was determined by optical microscopy and recorded.

### Human apoptosis proteome profiler array

To investigate the pathways by which HOXB13 induces apoptosis, a Proteome Profiler Array (RayBio Human Apoptosis Antibody Array Kit, RayBiotech, USA) was used to determine apoptosis-related protein levels according to the manufacturer’s instructions. In brief, 300 μg of proteins from HCT116/sh-HOXB13 and RKO/HOXB13 OE cells and the corresponding control cells were incubated with the human apoptosis array overnight. Apoptosis array data were quantified by scanning the membrane on a Biospectrum AC ChemiHR 40 (UVP, Upland, CA), and analysis of the array image file was performed using image analysis software according to the manufacturer’s instructions. Gene Ontology (GO) and Kyoto Encyclopedia of Genes and Genomes (KEGG) pathway enrichment analyses of the DEGs were conducted using a cluster profile. A *P*-value < 0.05 was selected as the cut-off criterion.

### Methylated DNA immunoprecipitation (MedIP) analysis

Methylated DNA immunoprecipitation (MedIP) was performed with a kit following the manufacturer’s instructions (D5101; Zymo Research). Briefly, genomic DNA was isolated from two pairs of tumor cells and sheared by sonication to generate fragments between ~200 bp and 500 bp in size. Then, sonicated DNA was immunoprecipitated with anti-5-methylcytosine monoclonal antibody to separate methylated DNA from unmethylated fragments. Enrichment in the MedIP fraction was measured by PCR. Sequences of the primers used to detect methylated DNA are listed in Supplementary Table 1.

### Multiplex immunohistochemistry (mIHC)

A TMA containing 56 CRC patient tissue samples was obtained. mIHC was performed to visualize the expression of HOXB13, DNMT3B and C-myc in the TMA according to the Opal immunostaining protocol. Briefly, the slides were deparaffined with xylene and ethanol, and heat-induced antigen retrieval was performed using microwave incubation. The sections were blocked in blocking buffer (Dako, X0909) for 10 min at room temperature. Then, the sections were incubated with primary antibody, HRP-conjugated secondary antibody and Opal working solution. Next, the sections were counterstained with DAPI. All the slides were scanned, and images were analyzed using inForm image analysis software (PerkinElmer).

### Statistical analysis

All the data were processed with SPSS statistical software (version 21.0; IBM Corp., Armonk, NY, USA) and are presented as the mean ± standard error of the mean (SEM). The chi-square test and Student’s *t*-test were performed for data comparisons. KM analysis and the log-rank test were performed for survival analysis. Pearson’s correlation analysis was used to analyze associations between gene expression levels. Differences for which *P* < 0.05 were considered to be statistically significant. The results are presented using GraphPad Prism (version 7.0; GraphPad Software, Inc., La Jolla, CA, USA).

## Supplementary information

Revised supplementary figure
