# Supplementary material for: Tumor-suppressive function and mechanism of HOXB13 in right-sided colon cancer
Source: Signal Transduct Target Ther. 2019 Nov 29;4:51. doi: 10.1038/s41392-019-0086-1 (PMC6882800; doi:10.1038/s41392-019-0086-1)
Supplement: Supplementary file 1 — Revised supplementary figure [file 41392_2019_86_MOESM1_ESM.pdf]

**Supplementary Information**

**(This file contains supplementary figures 1-9, supplementary table 1.)**

# Tumor suppressive function and mechanism of HOXB13 in right-sided colon cancer

Binbin Xie,[1#] Bingjun Bai,[2#] Yuzi Xu,[3] Yunlong Liu,[1] Yiming Lv,[2] Xing Gao,[4] Fei Wu,[5] Zhipeng Fang,[1] Ying Lou,[1] Hongming Pan, [1,*] and Weidong Han, [1,*]

[1] Department of Medical Oncology; Sir Run Run Shaw Hospital; School of Medicine, Zhejiang University, Hangzhou, 310016, PR China.

[2] Department of Colorectal Surgery; Sir Run Run Shaw Hospital; School of Medicine, Zhejiang University, Hangzhou, 310016, PR China.

[3] Department of Stomatology; Stomatology Hospital; School of Medicine, Zhejiang University, Hangzhou, 310000, PR China.

[4] Department of Medical Oncology; The Second Affiliated Hospital of Suzhou University; School of Medicine, Suzhou University, Suzhou, 215000, PR China.

[5] School of Medicine, Anhui University Of Science and technology, Huainan，232001，PR China

*Corresponding Author: Weidong Han, Sir Run Run Shaw Hospital, School of Medicine, Zhejiang University, 3# East Qinchun Road, Hangzhou, Zhejiang, China, 310016. Phone: +86-571-86006926; E-mail: hanwd@zju.edu.cn; and Hongming Pan, Sir Run Run Shaw Hospital, School of Medicine, Zhejiang University, 3# East Qinchun Road, Hangzhou, Zhejiang, China, 310016. Phone: +86-571-86006926; E-mail: panhongming@zju.edu.cn.

[#]These authors contributed equally to this work.

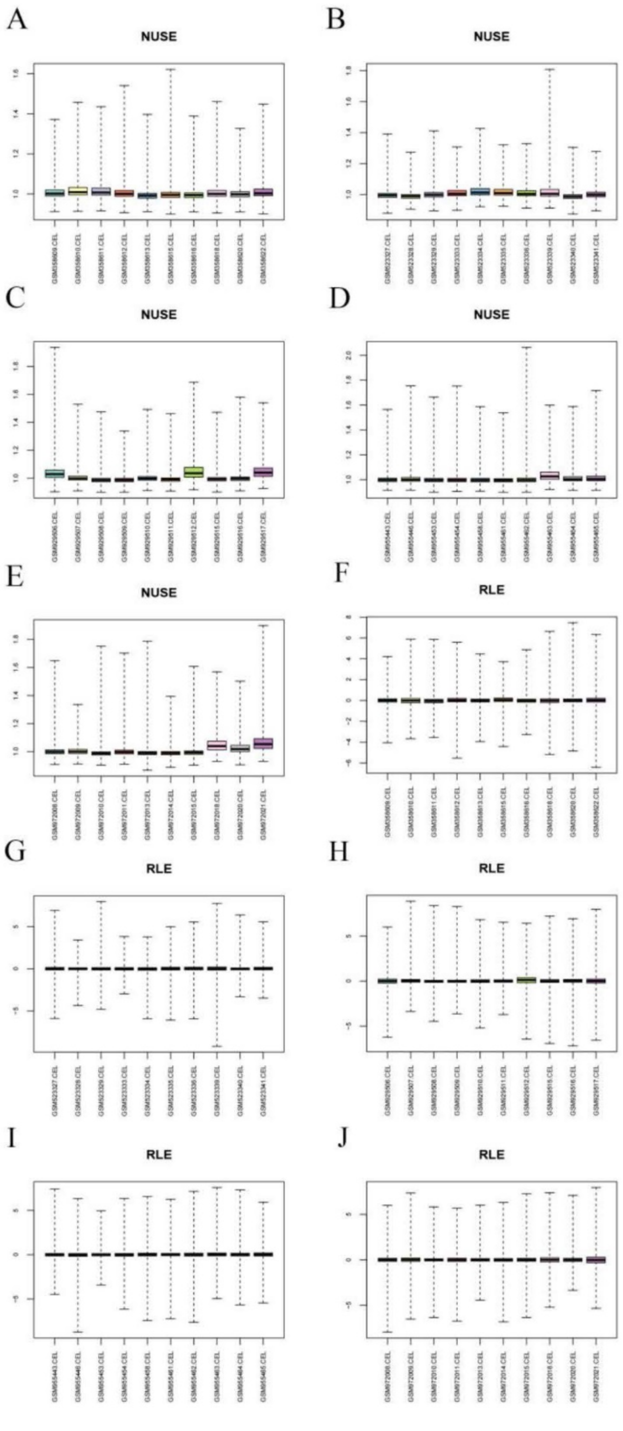


Supplementary Figure 1. Quality assessment of the five datesets. (A-E) Normalized unscaled standard error plots and (F-J) relative logarithmic analyses of GSE14333, GSE20916, GSE37892, GSE39084 and GSE39582.

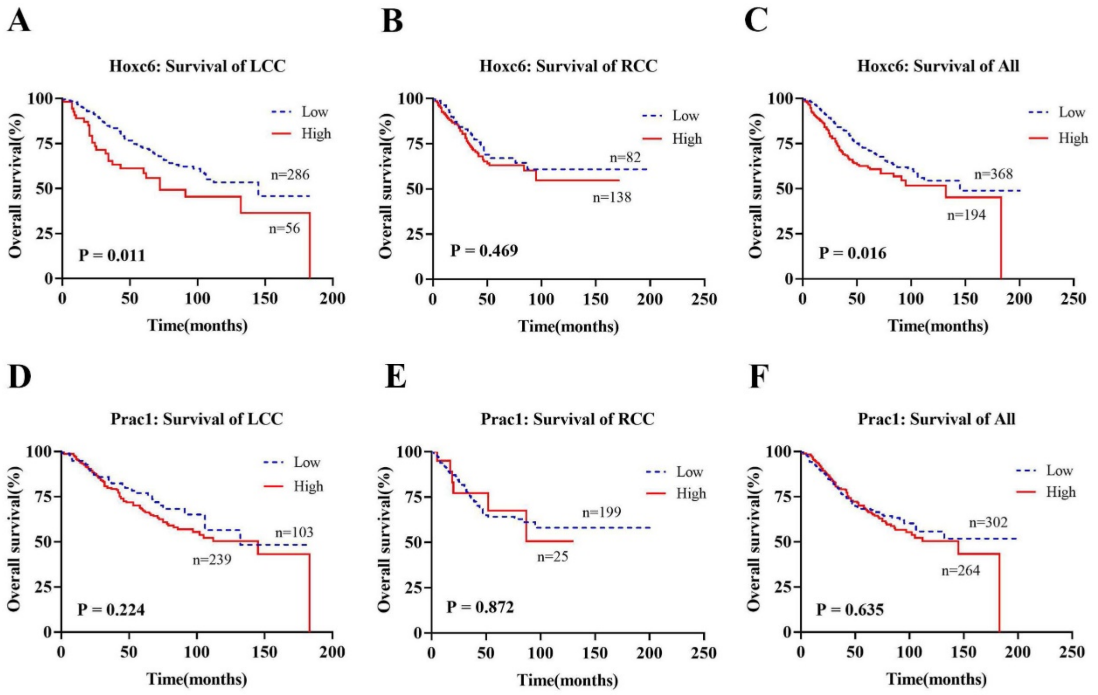


Supplementary Figure 2. Overall survival analyses according to tumor location. Survival analyses were conducted in LCC patients, RCC patients and all the CRC patients according to the expression levels of (A-C) HOXC6 and (D-F) PRAC1.

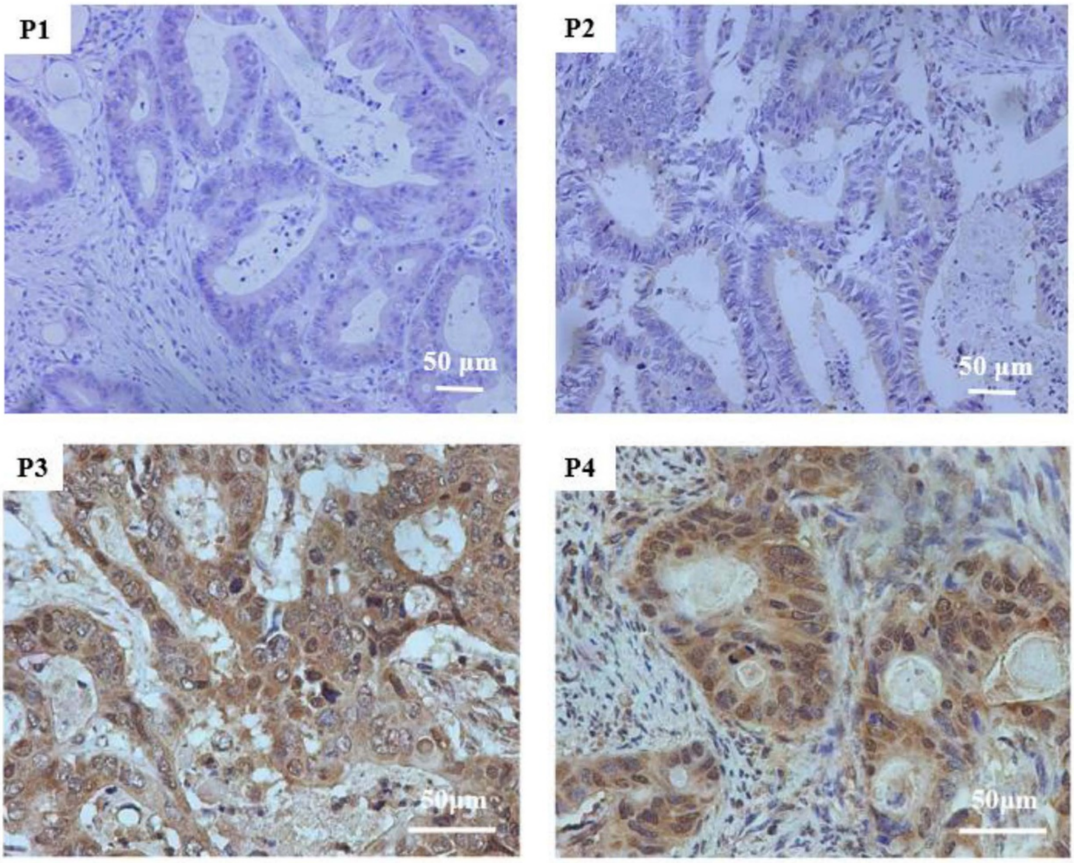


Supplementary Figure 3. Antigen-depletion of HOXB13 by IHC. (P1 and P2) Representative images of negative HOXB13 staining in tumor tissues. (P3 and P4) Representative images of HOXB13 staining in tumor tissues.

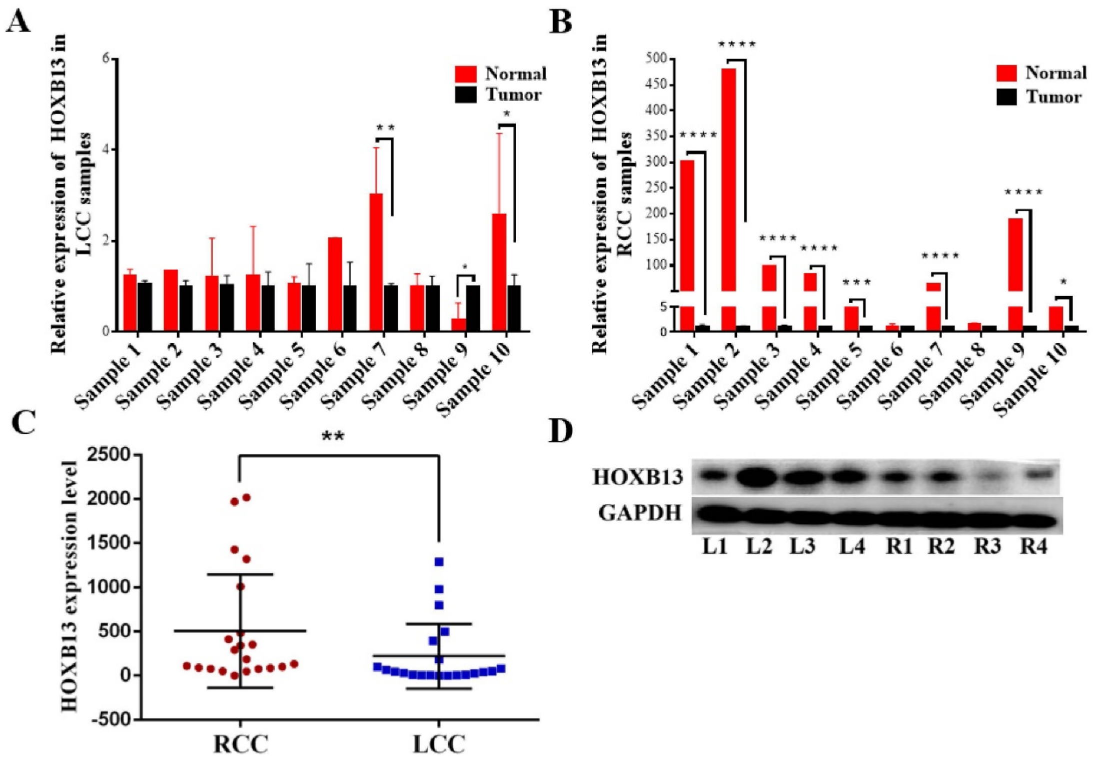


Supplementary Figure 4. HOXB13 expression in human tissues. (A, B) mRNA levels of HOXB13 expression in tumor and normal tissues from LCC and RCC patients. (C) The mRNA expression levels of HOXB13 in LCC (n=21) and RCC (n=21). (D) Protein expression levels of HOXB13 in LCC and RCC were examined by western blot analysis. GADPH was employed as a control. *P<0.05, **P<0.01, ***P<0.001, ****P<0.0001

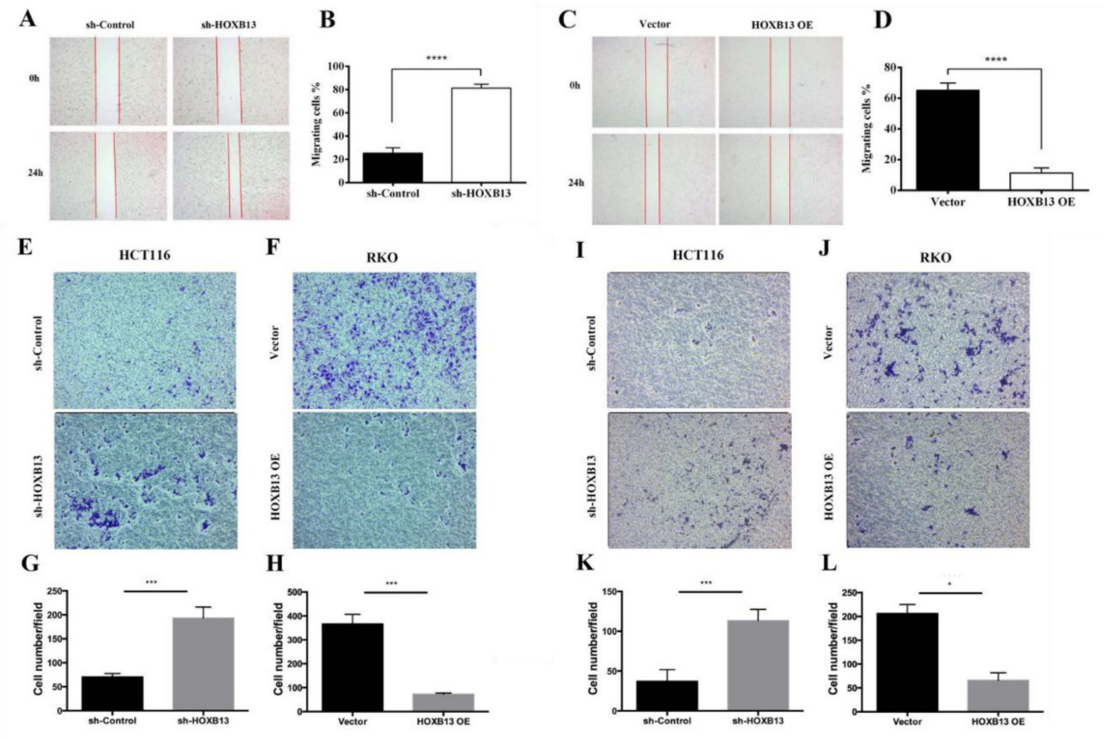


Supplementary Figure 5. HOXB13 inhibited cell migration. (A-D) A wound-healing assay was performed to detect the migration of sh-HOXB13 and HOXB13 OE cells. Transwell assays and quantitative analysis were used to further investigate the effect of HOXB13 on (E-H) cell migration and (I-L) cell invasion. *P<0.05, ***P<0.001.

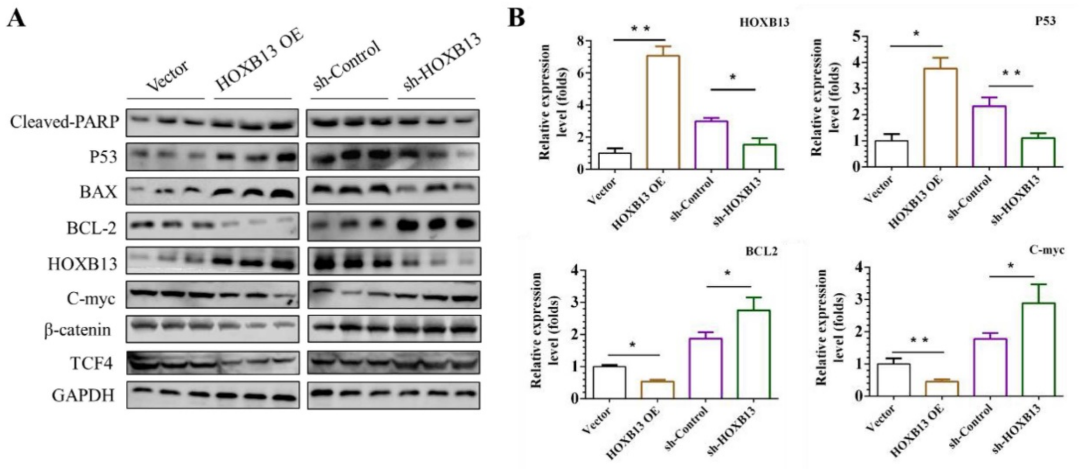


Supplementary Figure 6. HOXB13 regulates apoptosis-related genes in tumor tissues from nude mice. (A) Western blot and (B) QPCR were performed to detect the differentially expressed proteins regulated by HOXB13 in tumor tissues from nude mice. *P<0.05, **P<0.01

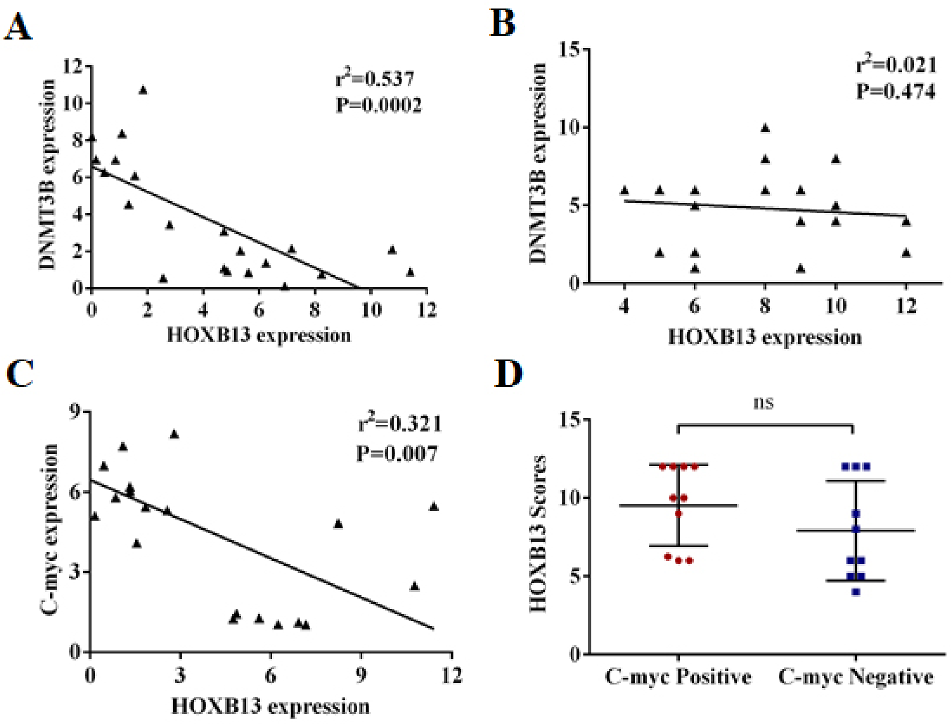


Supplementary Figure 7. The relationship between HOXB13, DNMT3B and C-myc expression. Quantitative analysis of correlation between HOXB13 and DNMT3B expression in (A) RCC according to QPCR (n=21) and (B) LCC (n=28) according to IHC staining score. (C) Comparison of HOXB13 and C-myc expression based on the mRNA levels in RCC (n=21). (D) Comparison between HOXB13 and C-myc expression according to IHC scores in RCC (n=33).

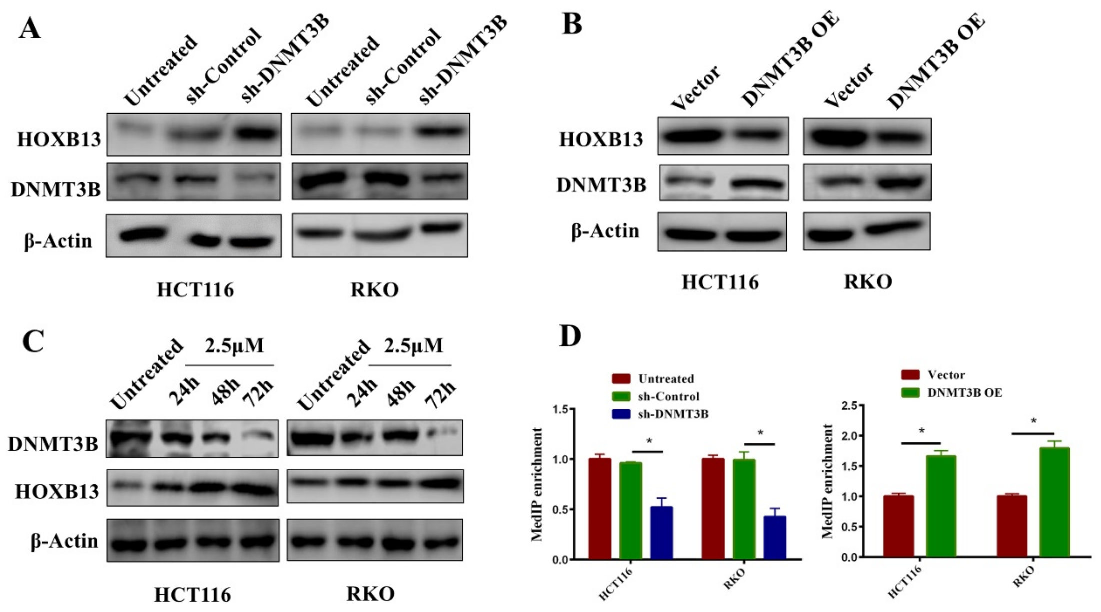


Supplementary Figure 8. DNMT3B regulates HOXB13 expression and methylation status. (A, B) HOXB13 knockdown and overexpression in HCT116 and RKO cells were validated by western blot analysis. (C) DNMT3B and HOXB13 were detected in cells treated with 2.5μM decitabine for 24h, 48h and 72h. (D) The methylation of the HOXB13 promoter in transfected HCT116 and RKO was analyzed by MedIP. *P<0.05

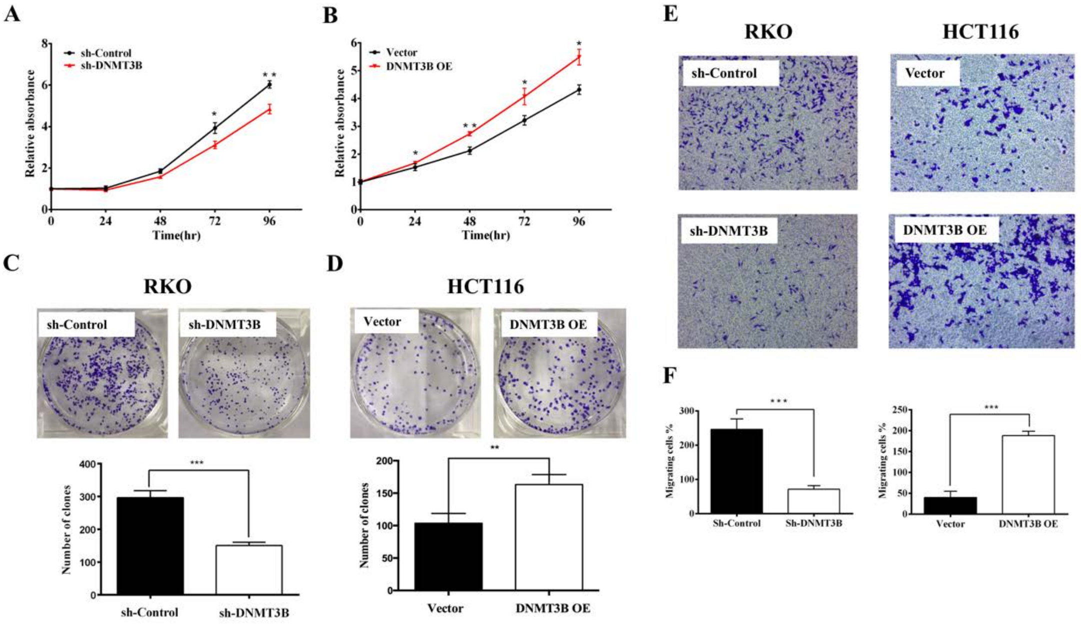


Supplementary Figure 9. DNMT3B promotes colon cancer cell vitality. (A, B) Proliferation of RKO/sh-DNMT3B and HCT116/DNMT3B OE cells were tested by CCK-8. (C, D) The capacity of colony formation was measured by clonogenic assay in RKO/sh-DNMT3B and HCT116/DNMT3B OE cells. (E, F) The effect of HOXB13 on cell migration was investigated by transwell assays and quantitative analysis. Data are presented as the mean ± SD. ***P <0.01. A t test was used for the statistical analysis.

**Supplement table 1.** Primers used in the study

| Genes | Real time PCR primers sequence |
|---|---|
| HOXB13 | F: 5’- CCAGTTACCTGGACGTGTCTG -3’ |
| | R: 5’- GGACCTGGTGGGTTCTGTTC -3’ |
| DNMT3B | F: 5’- ACCTCGTGTGGGGAAAGATCA-3’ |
| | R: 5’- CCATCGCCAAACCACTGGA-3’ |
| C-myc | F: 5’- TCTCCGTCCTCGGATTCTCT-3’ |
| | R: 5’- TTCTTGTTCCTCCTCAGAGTCG-3’ |
| β-actin | F: 5’-ACTCTTCCAGCCTTCCTTCC-3’ |
| | R: 5’-CGTCATACTCCTGCTTGCTG-3’ |
| Med IP | F: 5’- GCTCCAGTTGGGAGAACGAA-3’ |
| | R: 5’- CCTGTGACCTTTACAGCCCG-3’ |
